# The Seroprevalence of SARS-CoV-2 Antibodies among HealthCare Workers in University Hospital in Krakow before the Era of Vaccination

**DOI:** 10.3390/ijerph19074044

**Published:** 2022-03-29

**Authors:** Barbara Żółtowska, Ilona Barańska, Estera Jachowicz, Wojciech Sydor, Barbara Maziarz, Krzysztof Mydel, Anna Różańska, Barbara Wizner, Jerzy Rosiński, Magdalena Kossowska, Kaja Głomb, Jadwiga Wójkowska-Mach

**Affiliations:** 1 Center for Innovative Therapy, Clinical Research Coordination Center, University Hospital in Krakow, 30-688 Krakow, Poland; bzoltowska@su.krakow.pl (B.Ż.); wojciech.sydor@uj.edu.pl (W.S.); 2 Laboratory for Research on Aging Society, Department of Sociology of Medicine, Chair of Epidemiology and Preventive Medicine, Medical Faculty, Jagiellonian University Medical College, 31-034 Krakow, Poland; ilona.baranska@uj.edu.pl; 3 Chair of Microbiology, Medical Faculty, Jagiellonian University Medical College, 31-121 Krakow, Poland; estera.jachowicz@doctoral.uj.edu.pl (E.J.); jadwiga.wojkowska-mach@uj.edu.pl (J.W.-M.); 4 Department of Rheumatology and Immunology, Jagiellonian University Medical College, 30-688 Krakow, Poland; 5 Department of Clinical Biochemistry, Jagiellonian University Medical College, 31-066 Krakow, Poland; barbara.maziarz@uj.edu.pl; 6 Deputy Director for Coordination and Development, University Hospital in Krakow, 30-688 Krakow, Poland; krzysztofmydel@gmail.com; 7 Department of Internal Medicine and Gerontology, Jagiellonian University Medical College, 30-688 Krakow, Poland; barbara.wizner@uj.edu.pl; 8 Faculty of Management and Social Communication, The Institute of Economics, Finance and Management, Jagiellonian University, 30-348 Krakow, Poland; jerzy.rosinski@uj.edu.pl (J.R.); magdalena.kossowska@uj.edu.pl (M.K.); 9 Faculty of Management and Social Communication, The Institute of Applied Psychology, Jagiellonian University, 30-348 Krakow, Poland; kaja.glomb@uj.edu.pl

**Keywords:** seroprevalence, healthcare workers, COVID-19, infection prevention and control practices, work culture

## Abstract

Background: Knowledge of occupational health is crucial to the safety of healthcare workers in the pandemic period. The aim of our study was the rating of SARS-CoV-2 seroprevalence in connection with selected demographic, social, and organizational factors, as well as the identification of key elements determining the safety of HCWs and patients of the University Hospital in Krakow. Methods: This was a non-interventional, uncontrolled, open, single-center, cross-sectional online survey on the preparedness for the COVID-19 epidemic and the seroprevalence of medical and non-medical HCWs and students. Serum specimens from 1221 persons were tested using an immunoassay analyzer based on the ECLIA technique for the anti-SARS-CoV-2 antibodies IgM + IgG. Results: The total seroprevalence was 42.7%. In medical students it was 25.2%, while in physicians it was 43.4% and in nurses/midwives it was 48.1%. Of those who tested positive, 21.5% did not know their serological status. The use of personal protective equipment did not have any significant impact on the result of testing for anti-SARS-CoV-2 antibodies. The risk of developing the disease was not influenced by sex, professional work experience, workplace, or intensity of contact with the patient. Among the studied elements, only care of COVID-19 patients significantly increased the risk. The protective factor was starting work between the waves of the epidemic (June–September 2020). Conclusions: PPE is only one element of infection prevention and control—without other components, such as hand hygiene, it can be dangerous and contribute to self-infection. It is also very important to test healthcare workers. Not being aware of the COVID-19 status of HCWs poses a threat to other staff members, as well as patients and the family and friends of the infected. Thus, extreme caution should be applied when employing respirators with exhalation valves during the COVID-19 pandemic.

## 1. Introduction

Occupational health and safety in healthcare workers (HCWs) involves maintaining a physical distance of at least one meter from other people (including patients), patient cohorting and controlling the sources of microbes in patients, wearing a fitted half-face filtering mask during aerosol-generating procedures, and performing regular hand hygiene. However, while caring for coronavirus disease 2019 (COVID-19) patients, adequate personal protective equipment (PPE) should be employed, including a medical/surgical mask, face shield or goggles for eye protection, gown, and gloves [1]. It is also vital to comply with the relevant standards and transmission-based precautions.

A study conducted by the authors [2] on the preparedness of HCWs and medical students at the University Hospital in Krakow (UHK) for the COVID-19 pandemic pointed to a problem with the implementation of effective training. It also highlighted an unsatisfactory level of knowledge in the field of infection prevention and control in some groups of healthcare staff. The quoted results are based on a survey in which the degree of subjectivity of the respondents’ answers is difficult to assess when considering only the answers given. Connecting the answers of the respondents regarding their knowledge and compliance with the isolation precautions with independent variables—such as workplace, age, or the availability of personal protective equipment—and the seroprevalence of severe acute respiratory syndrome coronavirus 2 (SARS-CoV-2) antibodies, may provide more accurate data on the effectiveness of training and determinants of the application of isolation rules by healthcare professionals to ensure the safety of patients and the personnel themselves.

In COVID-19 disease, four types of diagnostic tests are used to assess the patient’s seroprevalence: enzyme-linked immunosorbent assay (ELISA) tests, rapid serology tests (RSTs), neutralization assays, and electrochemiluminescence immunoassay (ECLIA) tests. Unfortunately, each of the abovementioned methods has its limitations. In qualitative tests using ECLIA, RSTs, and neutralization assays, the result depends on whether patients’ antibodies are able to inhibit viral growth. Additionally, in RSTs, the result is dependent on the amount of antibodies in the patient’s serum. Chemiluminescent immunoassay may miss antibodies to viral proteins that are not involved in replication [3].

The aim of our study was to assess the seroprevalence of SARS-CoV-2 in medical staff at the UHK by selected demographic, social, and organizational factors in order to identify key elements determining the safety of staff and patients. The study was conducted after the big wave of COVID-19 in Poland in November 2020, when the highest 14-day rate of reported COVID-19 cases per 100,000 population was 877 (week 46 of 2020). In Poland, from 4 March—when the first case of COVID-19 was detected—until 31 December 2020, a total of 1,322,947 cases were reported, including 87,471 infections of healthcare workers, accounting for 6.6% of all infections [2].

## 2. Methods and Materials

### 2.1. Study Design

This was a non-interventional, uncontrolled, open, single-center, cross-sectional online survey on the preparedness for the COVID-19 epidemic and the seroprevalence of medical and non-medical staff of the UHK as well as medical students who underwent clinical rotations at the UHK in the autumn of 2020, as a part of the CRACoV-HHS study (CRACOW in COVID-19 pandemic—Hospital, Home, and Staff) [4].

### 2.2. Serologic Testing, Data Collection, and Data Compilation

The epidemiological part of the study was based on the modified World Health Organization (WHO) *Assessment of Risk Factors for Coronavirus Disease 2019 (COVID-19) in Health Workers: Protocol for a Case–Control Study* (Supplementary Materials). The questionnaire was slightly modified. Modifications included the addition of some more detailed information in the demographic part of the questionnaire—e.g., the experience of work in healthcare in years—the type of ward (unit in hospital), and removing the question on nationality. Some other questions was deleted in the part concerning exposure to COVID-19-infected patients—that is, questions number 4, 5, and 16 from part 5 in the original questionnaire. Instead, in part 4 of this questionnaire, two questions on subjective assessment of knowledge of effectiveness of training were added. These were: “Do you think that you were sufficiently trained on the prevention patients and yourself of infections?” and “Are you able to properly follow the infection protection procedures?” The study was carried out in parallel with the vaccination campaign. Vaccination earlier than 4 days before blood donation was the exclusion criterion for the participants. The methods and studied population have previously been described in detail [2].

Serum specimens from 1221 persons were tested. Venous blood samples were collected using a serum separator tube. Tubes were centrifuged at 4000× *g* for 15 min to separate the serum. All antibody tests were performed using a commercially available fully automated Roche Cobas e801 immunoassay analyzer based on the electrochemiluminescence “ECLIA” technique (Elecsys^®^ Anti-SARS-CoV-2, Roche, Mannheim, Germany), with specificity and sensitivity of 99.06% and 89.5%, respectively [5]. The anti-SARS-CoV-2 S is an immunoassay for the in vitro quantitative determination of antibodies (including IgG) to the SARS-CoV-2 spike (S) protein receptor-binding domain (RBD) in human serum and plasma. The assay uses a recombinant protein representing the RBD of the S antigen in a double-antigen sandwich assay format, which facilitates the detection of high-affinity antibodies against SARS-CoV-2. The test is intended as an aid to assess the adaptive humoral immune response to the SARS-CoV-2 S protein. The calibration and internal quality control was performed according to the manufacturer’s recommendations. Patient samples were labelled as reactive (cutoff > 1.000) and non-reactive (cutoff < 1.000), as recommended by the manufacturer.

### 2.3. Study Participants

The study was carried out in the UHK—the largest teaching hospital in southern Poland—with 39 clinical departments (1310 beds in total), 2 intensive care departments (40 beds), 7 institutes, and 68 outpatient clinics; the amount of full-time equivalent (FTE), physicians and nurses (including midwives) are 873 and 1654, respectively, per 1310 beds. The participants were investigated between 4 and 19 January, 2021. Participation in the study was voluntary. The participants were eligible for enrolment if they met the following inclusion criteria: (1) hospital employee or (2) fifth-year medical student, (3) voluntary consent to participate in the study. The sole key exclusion criterion was refusal to participate in the study and receiving the COVID-19 vaccine no earlier than 4 days before the day of collecting the material (blood sample) for testing.

The participants were divided into 5 professional categories based on their level of training in infection prevention and control, and according to standards of pre- and postgraduate education:

- Physicians;
- Fifth-year medical students;
- Nurses/midwives;
- Other HCWs (e.g., healthcare assistant, radiology technician, physiotherapist/rehabilitation specialist, dietician, psychologist, social worker, volunteer, cleaning staff, diagnostic laboratory technician, registration/patient information desk worker);
- Administrative staff.

The fifth-year students only were invited to the study because of their participation in clinical rotations at the UHK during the pandemic and completion of their entire pre-graduate medical education. In addition, fifth-year students were invited to volunteer at the UHK and other hospitals [2].

The study was conducted according to the guidelines of the Declaration of Helsinki, and approved by the Bioethics Committee of the Jagiellonian University (protocol code 1072.6120.353.2020, date of approval 16 December 2020). All data entered into the electronic database and analyzed in this study were anonymized.

### 2.4. Statistical Analysis

The association between the incidence of SARS-CoV-2 infection and selected variables (e.g., age, occupation, work experience, workplace, direct contact with patients—with or without COVID-19—as first-line HCWs) was studied using the chi-squared test and the *t*-test for independent samples on the complete series of cases (*n* = 1221 individuals) (Table 1). The analysis was carried out on a sample of all employees included in the study who had done the serological test (*n* = 1221). Next, using the chi-squared test on a subset of individuals who had been in direct contact with patients with or without COVID-19 (*n* = 719), the association was assessed between the compliance with chosen elements of prevention and control of infections and the occurrence of a positive result of the test for SARS-CoV-2 (Table 2). The analysis was conducted on a group of people who had been in direct contact with patients, with or without COVID-19 (i.e., physicians, nurses, care providers, technicians, physiotherapists, paramedics, cleaning staff; *n* = 719). Table 3 presents the relationship between the occupation and the workplace, and the declaration that SARS-CoV-2 infection was the result of occupational exposure. To that end, the chi-squared test was applied and the calculations were carried out on a group of 223 workers who were aware of SARS-CoV-2 infection (data coming from a survey questionnaire) and had been in direct contact with patients with or without confirmed COVID-19 status. Two multivariable logistic regression models were constructed: The first of them (Table 4) concerned the relationship between SARS-CoV-2 infection and selected factors (i.e., occupation, workplace, work in a unit with patients infected with COVID-19, and a declaration that the respondent always applies hand hygiene in accordance with WHO recommendations). The analysis was carried out on a group of people who had been in direct contact with patients with or without COVID-19 (*n* = 719). The second logistic regression model (Table 5) concerned the evaluation of 416 individuals to determine what factors were associated with the respondents not being aware of their SARS-CoV-2 infection (i.e., in the questionnaire, they indicated that they were not infected, while the serological test result indicated that they had in fact been infected). Multivariable analyses were preceded by univariable analyses. To build the multivariable models, we used a stepwise backwards elimination procedure. Results for multivariable logistic models are presented as odds ratios (ORs) and 95% confidence intervals (95% CIs). An alpha level of < 0.05 was adopted for the analyses to ascertain the statistical significance of the effect. Analyses were performed using the IBM SPSS Statistics 26 package for Windows (IBM SPSS Statistics, IBM Corporation, Chicago, IL, USA).

## 3. Results

### 3.1. Descriptive Characteristics of the Study Population

Altogether, 1221 people working at the UHK were subjected to SARS-CoV-2 testing. A positive result was demonstrated for 521 (42.7%) of them. This was significantly more common in physicians (43.3%) and nurses (48.0%), while the students were sick less often (25.2%).

People who underwent training in infection prevention and control (all infections—not just COVID-19) fell ill significantly more often than people without this type of training (44.5% vs. 33.5%).

People aged 37–55 years were the most vulnerable to being infected. The risk of developing the disease was not influenced by sex, seniority in the profession (excluding the student group), workplace, or intensity of contact with the patient—regardless of whether the patient had COVID-19 or other illnesses. The examined non-professional social situations (e.g., public transport) also showed no association with a positive SARS-CoV-2 test result (Table 1). Out of the 521 people with a positive test result, 189 (21.5%) did not know their own status.

### 3.2. Relationship between a Positive Result for COVID-19 and Selected Elements of Prevention and Control of Infections

All participants—with or without COVID-19—who had direct contact with the patients reported the use of proper PPE, but that use was not significantly associated with SARS-CoV-2 IgM + IgG. In other words, the seroprevalence was 48.2% among those using PPE and 42.9% among those not using PPE or who reported “I don’t know” (*p* = 0.066); seroprevalence was 47.7% among those using a surgical mask and 56.3% among those using a filtering half-mask with diagnostic gloves (*p* = 0.349). In the questionnaires (self-evaluation), the respondents fully confirmed the availability of PPE and their ability to use it at the UHK. Selected elements of the prevention and control of infections among HCWs (e.g., awareness of the “moments for hand hygiene” recommended by the WHO or preferred hand hygiene techniques) were not associated with a positive SARS-CoV-2 test result (Table 2).

### 3.3. Relationship of Occupational Exposure in People with a Positive Result for COVID-19

When analyzing the results of the questionnaire survey regarding the results of the antibody test, it was found that out of 223 HCWs who knew that they had COVID-19 (regardless of the severity of the symptoms), 117 people declared occupational exposure. Out of 223 HCWs who were aware of the SARS-CoV-2 infection, only 27 (12.1%) people stated that their illness was not related to occupational exposure, and a further 79 people did not know whether there was a connection between their duties and the disease (Table 3). Physicians significantly more often did not know how to classify their case (professional exposure, yes or no?): 13 (68.4%) vs. 61 (33.9%) nurses/midwives and 5 (20.8%) other HCWs (*p* = 0.023) (Table 3). Only 19 (39.6%) ICU or ED workers and 78 (56.1%) non-ICU workers stated that their illness was related to occupational exposures; 41.7% of operating room workers and 29.2% of other workers reported that they did not know how to classify their illnesses (Table 3).

### 3.4. Multivariable Analysis of Factors Associated with the Course of the Infection, i.e., a Positive Test Result for SARS-CoV-2

It was demonstrated that among the people who were caring for COVID-19 patients (or were coming into contact with them), nurses were 1.7 times more likely to become infected in comparison to other HCWs (OR 1.7, 95% CI 1.02–2.73, *p* = 0.042), and people working in non-ICU/ED units were 1.5 times more likely to get infected compared to ICU/ED workers (OR 1.5, 95% CI 1.05–2.26, *p* = 0.027). The other results were not statistically significant (see Table 4).

### 3.5. Multivariable Analysis Describing Respondents with Asymptomatic Course of Infection Who Were Not Aware of It

In the group of people with a positive test result (*n* = 521), 189 people (36.3%) did not know that they had been infected with the disease, i.e., its course was asymptomatic or oligosymptomatic. In the analysis of factors affecting the lack of knowledge about past infection—i.e., with a positive serological test result, the respondent indicated the absence of the disease—it was found that people caring for patients with COVID-19 were significantly less often aware of the infection they had suffered from (OR 2.3, 95% CI 1.32–3.90, *p* = 0.003), while employees who started their work with patients with confirmed infections in the period between the second and third waves of infection—i.e., between June and September 2020—knew their serological status significantly more often (OR 0.5, 95% CI 0.29–0.96, *p* = 0.035). Students were significantly (fivefold) less likely to know their serological status compared to other HCWs (OR 4.9, 95% CI 1.43–16.73, *p* = 0.012). Age was also significant, as among the people aged 37–55, not knowing their serological status was significantly more rare (OR 0.7, 95% CI 0.36–0.95, *p* = 0.031). All results are presented in Table 5.

## 4. Discussion

The seroprevalence observed in the study (43%) was several times higher than that reported in other countries: 11.2% in Sweden, June 2020 [6]; 12.2% in Italy, May 2020 [7]; 3.8% in Atlanta, USA, June 2020 [8] and USA, May 2020 [9]; or 4.0% in Denmark, October 2020 [10]. This situation is not directly related to the incidence in the general population, as in December 2020 (right before our study began) cumulative incidence in Poland was 299 cases per 10,000 population—similar to that in Sweden (313) or in Italy (305), while in the USA it was much higher (494) and for Denmark much lower (189 cases per 10,000 population) [11].

To some extent, the obtained results may be explained by the potentially greater intensity of the contact between HCWs and patients in Polish hospitals than in the countries from which the cited results come. According to the data from the Organization for Economic Co-operation and Development (OECD),the number of nurses per 100 hospital beds in Sweden is 5.2; in Denmark it is 3.9, and in Italy it is 1.95, while in Poland it is five times less, at 0.78 [12]. At the UHK, this ratio amounted to 0.9, and it was similar to the one obtained in the study by Różańska et al. concerning occupational exposure in five hospitals in Małopolska, i.e., amounting to 0.93 [13]. This study reported a slightly lower ratio of doctors per 100 beds than at the UHK, amounting to 33.62 and 52.4, respectively, but the index expressing the number of physicians in relation to the number of beds in Poland is 0.4, and this is lower than the corresponding values of the indicators in Sweden, Italy, and Denmark, amounting to 2.1, 1.3, and 1.6, respectively. Moreover, these data can confirm the connection between the intensity of conducting diagnostic and therapeutic procedures while working with patients—higher in Poland than in most other European countries, and higher at the UHK among nurses than among doctors. Staff shortages, however, do not fully explain the results obtained, as the basis for the safety of medical workers should be the PPE available according to the requirements, together with its correct use.

The results of our study show that HCWs who underwent training in infection prevention and control (all infections, not just COVID-19) were sick significantly more often than those without this type of training. The reasons for such a state can be both systemic and individual. The systemic reasons include the possibility that people after training were directed to more burdensome and difficult tasks compared to people without training. For the individual reasons, it is worth considering the so-called heuristics of the optimistic future: people after training may have subjectively estimated their risk of infection as lower, because compared to others they perceived themselves as better protected against infection.

On the other hand, the fact that nurses were 1.7 times more likely to be infected could be related not to their knowledge of protective measures, but to the number of hours per day in close contact with COVID-19 cases. Their contact for that reason is usually longer compared to that of doctors or medical students.

Unfortunately, our study demonstrated that the use of PPE did not constitute a protective factor, and additionally pointed to significant gaps in knowledge of the basics of IPC. Almost half of the respondents who had direct contact with patients, with or without COVID-19, were not able to indicate the correct number of “moments for hand hygiene”, and either did not apply the standard (or transmission-based) precautions or did not know them. We are therefore dealing with a classic case of the difference between a declared attitude (“Yes, I follow the rules”) and the actual behavior (lack of knowledge and/or lack of skills in applying hygiene standards—especially in the field of handwashing) [14].

Our observations are not surprising, as research from 2016 to 2018—from before the COVID-19 pandemic—concerning the factors that determine the perception and application of the principles of standard isolation in Polish hospitals, demonstrated a significant problem related to their implementation and the lack of their satisfactory acceptance by medical personnel [15]. Other studies in this respect, concerning Polish medical staff in the period before the COVID-19 pandemic, showed an unsatisfactory level of knowledge regarding the basics of hand hygiene, but also a possibly culturally conditioned tendency to disregard the rules and apply them at will [16]. This points to the possibility that at least one of the three points concerning the competence transfer from the training to the workplace may not function well enough. These three elements are the training itself (i.e., teaching how to perform activities, indicating their significance in every day functioning in the workplace), the period of up to 2 weeks after training, during which the skills are implemented as habits of conduct learned during the training, and supervision of immediate superiors over the performance of activities learned during the training over a longer period of time (e.g., 6–12 months) in the workplace (ward) [17,18,19].

Additionally, the employment of PPE can be inconvenient, and obliges HCWs to comply with the IPC rules—especially proper hand hygiene. In the study by Peres et al., employees reported discomfort while wearing masks (including respirator masks), affecting task performance and communication, and causing dyspnea, skin rash, and headache. The most common mistakes involved touching the front of the mask while in use and omitting hand hygiene before or after the use of a mask [20]. Therefore, it should be remembered that PPE is only one of the elements of infection prevention and control, and without hand hygiene, for example, using PPE can be dangerous and contribute to self-infection. Another issue is whether perceiving the compliance with hygiene rules as inconvenient is associated with lower effectiveness (mentioned above) of the three critical points for the transfer of competences from training to behavior at the workplace—especially in the context of the previously described lack of knowledge regarding hand hygiene [2].

Our study demonstrated that 36.3% of infected personnel had a mild or asymptomatic course of COVID-19 and were unaware of their infection. Regrettably, the research by Park et al. [21] and the results of meta-analyses [22] confirm a high proportion of oligosymptomatic and asymptomatic people in the COVID-19 pandemic, which may be up to one-quarter of all cases. In asymptomatic individuals, the SARS-CoV-2 virus can be isolated from saliva; therefore, there is a potential chance of viral transmission [23]. For this very reason, the National Institute for Occupational Safety and Health (NIOSH) of the United States Centers for Disease Control and Prevention (CDC) suggests extreme caution when employing respirators with exhalation valves. During the COVID-19 pandemic, medical professionals have expressed concern that healthcare personnel infected with SARS-CoV-2 may spread the disease from unfiltered exhaled air passing through their respirator’s exhalation valve [24]. On the other hand, the manufacturers of valved masks say that they provide respiratory protection only against dust and mist, so they are not recommended for work with patients or people susceptible to infections [25].

Facepiece filtering respirators with exhalation valves should also not be used in the community, so as to limit SARS-CoV-2 diffusion [26,27].

It is a very dangerous situation when HCWs do not know their COVID-19 status, as this means that they were probably performing their professional duties while infected with the disease. This highlights the importance of daily practices in infection control and prevention. Unfortunately, half of the surveyed medical workers (50.5%) from the UHK had a low or very low knowledge index regarding the “moments for hand hygiene”, “I always follow standard precautions”, and “I always use personal protective equipment according to the exposure assessment” [2].

Students seem to be another group that requires special attention in terms of training and compliance with the rules (Table 3). It was significantly more often that they were not aware of having suffered from an infection; in the studied wave of COVID-19, they went through it asymptomatically or with few symptoms. At the same time, their short work experience or completion of many training courses in a very short timeframe may be factors impacting their insufficient internalization of behaviors related to the prevention of infections.

In the studied population of medical workers, as many as 120 people declared occupational exposure, while in the hospital under study, according to internal registers from 2020, only 38 HCWs and cleaning staff reported occupational exposure [2]. Hence, it is quite probable that a substantial proportion of SARS-CoV-2 infections in the studied hospital were not described or analyzed. It is difficult to determine the reason for the failure to report and the lack of a prospective study of causes and countermeasures for the viral transmission, but this fact seems to correspond with what was previously described by the authors, i.e., a lack of positive perception of the work of the infection prevention and control team by medical staff not only at the UHK, but in Polish healthcare facilities in general [28].

## 5. Strengths and Limitations

To our best knowledge, the results presented here are the first study of SARS-CoV-2 seroprevalence outside the English-speaking countries conducted on a large sample of respondents, in the largest university hospital in Poland, where clinical training is regularly conducted; however, due to the nature of the survey, it was not conducted on a random sample. An assessment of the representativeness of the data for the staff of the entire hospital indicates an overrepresentation of nurses. Due to the intensity of staff responsibilities in relation to the care of COVID-19 patients, no objective survey (test) of knowledge was conducted, but only a subjective assessment of the training of the respondents. Unfortunately, the study mainly depended on a subjective questionnaire, which may have led to significant bias.

## 6. Conclusions

In the group of Polish HCWs under study, a very high level of SARS-CoV-2 seroprevalence was obtained, and over 20% of infections were found to have been asymptomatic. Significant factors of exposure were being a nurse and working in a non-ICU/ED unit. PPE is only one of the elements of infection prevention and control, and effective prevention of infection (including self-infection) demands knowledge of and compliance with hand hygiene recommendations. 

## Figures and Tables

**Table 1:** Characteristics of the study group (*n* = 1221).

Investigated Professional and Non-Professional Factors	Seroprevalence of SARS-CoV-2 Antibodies (*n* = 1221)			*p*-Value
	Yes *n* (%)	No *n* (%)	Total *n* (%)	
Sex				
Male	59 (38.8)	93 (61.2)	152 (12.4)	0.305
Female	462 (42.3)	607 (56.8)	1069 (87.6)	
Age				
Average, SD	42.0 (12.3)	40.6 (12.9)	41.3 (12.7)	0.071
18–36 years	178 (38.1)	289 (61.9)	467 (38.2)	0.017
37–55 years	270 (46.8)	307 (53.2)	577 (47.3)	
56 years or more	73 (41.2)	104 (58.8)	177 (14.5)	
Occupation				
Physicians	43 (43.4)	56 (56.6)	99 (8.1)	<0.0001
Medical students	30 (25.2)	89 (74.8)	119 (9.7)	
Nurses/midwives	305 (48.0)	331 (52.0)	636 (52.1)	
Other HCWs	79 (40.7)	115 (59.3)	194 (15.9)	
Administrative workers	64 (37.0)	109 (63.0)	173 (14.2)	
Work experience				
Less than 12 months	42 (37.5)	70 (62.5)	112 (9.2)	0.073
1–5 years	111 (38.3)	179 (61.7)	290 (23.8)	
6 years or more	368 (33.9)	451 (55.1)	819 (67.1)	
In the past 14 days, how often have you used public transport?				
Almost every day	117 (38.0)	191 (62.0)	308 (25.2)	0.224
Often	71 (41.8)	99 (58.2)	170 (13.9)	
Rarely	94 (43.7)	121 (56.3)	215 (17.6)	
I do not use public transport	239 (45.3)	289 (54.7)	528 (43.2)	
In the past 14 days, how often have you had social interaction with individuals outside of work or family?				
Almost every day	178 (39.9)	268 (60.1)	446 (36.5)	0.395
Often	181 (45.5)	217 (54.5)	398 (32.6)	
Rarely	155 (42.7)	208 (57.3)	363 (29.7)	
There was no such situation	7 (50.0)	7 (50.0)	14 (1.1)	
Where do you work?				
ICU with COVID-19	71 (44.1)	90 (55.9)	161 (13.2)	0.115
ICU without COVID-19	14 (29.2)	34 (70.8)	48 (3.9)	
Non-ICU with COVID-19	103 (50.0)	103 (50.0)	206 (16.9)	
Non-ICU without COVID-19	173 (43.4)	226 (56.6)	399 (32.7)	
ED, outpatient clinic	31 (38.3)	50 (61.7)	81 (6.6)	
Medical imaging	32 (39.0)	50 (61.0)	82 (6.7)	
Others	97 (39.8)	147 (60.2)	244 (20.0)	
Are you taking care of or do you have direct contact with a patient, with or without COVID-19?				
Yes	445 (43.5)	578 (56.5)	1023 (83.9)	0.183
No	76 (38.4)	122 (61.6)	198 (16.2)	
Are you taking care of or do you have direct contact with a COVID-19 patient?				
Yes	364 (44.0)	463 (56.0)	827 (67.7)	0.169
No	157 (39.8)	237 (60.2)	394 (32.3)	
Have you ever been positive for SARS-CoV-2?				
Yes	332 (97.4)	9 (2.6)	341 (27.9)	<0.001
No OR I do not know	189 (21.5)	691 (78.5)	880 (72.1)	
Did you receive infection prevention and control training (all infections, not just COVID-19)? *n* (%)				
Yes	452 (44.5)	563 (55.5)	1015 (83.1)	0.003
No	69 (33.5)	137 (66.5)	206 (16.9)	
Did you receive specific training in the care of COVID-19 patients?				
Yes	266 (43.6)	344 (56.4)	610 (56.5)	0.860
No	202 (43.1)	267 (56.9)	469 (43.5)	
Are you familiar with the “moments for hand hygiene” recommended by the WHO				
Correct answer	356 (43.2)	469 (56.8)	825 (67.6)	0.623
Incorrect answer OR I do not know them	165 (41.7)	231 (58.3)	396 (32.4)	

Legend: ED, emergency department; ICU, intensive care unit; WHO; World Health Organization.

**Table 2:** Relationship between a positive result for SARS-CoV-2 IgM + IgG and selected elements of prevention and control of infections in people who have direct contact with patients, regardless of COVID-19 (*n* = 719).

Selected Elements of the Prevention and Control of Infections in Healthcare Facilities	Seroprevalence of SARS-CoV-2 Antibodies (*n* = 719)		*p*-Value
	Positive (%)	Negative (%)	
Are you familiar with the “moments for hand hygiene” recommended by the WHO?			
Yes, all 5	266 (46.9)	301 (53.1)	0.291
I do not know them OR incorrect answer	64 (42.1)	88 (57.9)	
The preferred hand hygiene technique			
Hand rubbing	60 (49.6)	61 (50.4)	0.519
Others, hand washing with OR without hand rubbing	192 (46.3)	223 (53.7)	
Hand hygiene before contact with patient (1st“moment for hand hygiene”)			
Always	227 (48.6)	240 (51.4)	0.055
In most cases OR sometimes OR rarely OR never	25 (36.2)	44 (63.8)	
Do you follow IPC standard precautions when in contact with any patient?			
Always	232 (45.9)	273 (54.1)	0.226
In most cases OR sometimes OR rarely OR never	93 (47.7)	102 (52.3)	
I do not know what IPC standard precautions are	3 (23.1)	10 (76.9)	

Legend: IPC, infection prevention and control.

**Table 3:** Relationship of occupational exposure in people with a positive result for SARS-CoV-2 IgM + IgG performing various occupations at the UHK (*n* = 223).

Characteristics of People with a Positive Test Result for SARS-CoV-2 IgM + IgG	Did the Infection Result from Occupation Exposure? Self-Reported Declaration *n* = 223 (%)			*p*-Value
	Yes	No	I do not know	
Profession				
Physicians	5 (26.3)	1 (5.3)	13 (68.4)	0.023
Nurses/midwives	96 (53.3)	23 (12.8)	61 (33.9)	
Other HCWs	16 (66.7)	3 (12.5)	5 (20.8)	
Workplace restricted to COVID-19 cases				
ICU or ED	19 (39.6)	8 (16.7)	21 (43.8)	0.002
Non-ICU	78 (56.1)	15 (10.8)	46 (33.1)	
Operation room	6 (50.0)	1 (8.3)	5 (41.7)	
Others	14 (58.3)	3 (12.5)	7 (29.2)	

Legend: ED, emergency department; ICU (with COVID-19 and without), intensive care unit; HCW, healthcare worker.

**Table 4:** Relationship between the occurrence of SARS-CoV-2 infection and selected factors among the staff who had direct contact with patients with or without COVID-19—multivariable logistic regression analysis (*n* = 719).

Study Group/Profession (*n* = 719)	OR	95% CI	*p*-Value
I use proper hand hygiene according to the “WHO 5 moments”, always vs. others	0.9	0.71–1.32	0.852
Occupation			
Other HCWs	Ref.		
Physicians	1.2	0.60–2.26	0.654
Nurses/midwives	1.7	1.02–2.73	0.042
Workplace:			
ICU, ED	Ref.		
Non-ICU	1.5	1.05–2.26	0.027
Operation room	1.0	0.55–1.92	0.925
Outpatient clinic	0.6	0.55–1.47	0.285
Other	1.0	0.52–2.02	0.947
Working with COVID-19 patients, yes vs. no	0.0	0.68–1.36	0.812

Legend: ED, emergency department; ICU, intensive care unit; HCW, healthcare worker.

**Table 5:** Relationship between selected variables and the unawareness of the history of SARS-CoV-2 infection among the staff who had had asymptomatic infection (*n* = 416)—multivariable logistic regression analysis.

Study Group/Profession (*n* = 416)	OR	95% CI	*p*-Value
Sex, female vs. male	0.5	0.25–1.12	0.121
Age			
18–36	Ref.		
37–55	0.7	0.36–0.95	0.031
56 or more	0.7	0.36–1.47	0.368
Caring for a COVID-19 patient	2.3	1.32–3.90	0.003
Occupation			
Other HCWs	Ref.		
Physicians	2.5	0.97–6.35	0.58
Medical students	4.9	1.43–16.73	0.012
Nurses/midwives	1.5	0.69–3.29	0.303
Workplace			
Outpatient clinic	Ref.		
Non-ICU	0,6	0.18–1.78	0.325
ICU	0.5	0.13–1.76	0.275
Operation room	1.8	0.44–7.03	0.426
Other	0.6	0.16–2.52	0.519
When was the first known contact with a COVID-19 patient?			
February–May 2020	Ref.		
June–September 2020	0.5	0.29–0.96	0.035
October–December 2020	1.3	0.69–2.31	0.455
Infection training rating, high vs. low	1.0	0.58–1.90	0.881
Compliance with the standard precautions, always vs. not always	1.0	0.58–1.62	0.903
Proper application of IPC procedures, yes vs. no	0.8	0.28–2.48	0.752

Legend: IPC, infection prevention and control.

## Data Availability

The datasets generated or analyzed during this study are available and can be obtained, upon reasonable request, from Ilona Barańska (email: ilona.baranska@uj.edu.pl).

## Supplementary Materials

The following supporting information can be downloaded at: https://www.mdpi.com/article/10.3390/ijerph19074044/s1. File S1: Questionnaires and guidance.

## Abbreviations

95% CI, 95% confidence interval; COVID-19, coronavirus disease 2019; ECLIA, enzyme-linked immunosorbent assay; ED, emergency department; FTE, full-time equivalent; HCW, healthcare worker; ICU, intensive care unit; IPC, infection prevention and control; OR, odds ratio; PPE, personal protective equipment; RST, rapid serology test; SARS-CoV-2, severe acute respiratory syndrome coronavirus 2; UHK, University Hospital in Krakow; WHO, World Health Organization.
