# Supplementary material for: The Seroprevalence of SARS-CoV-2 Antibodies among HealthCare Workers in University Hospital in Krakow before the Era of Vaccination
Source: Int J Environ Res Public Health. 2022 Mar 29;19(7):4044. doi: 10.3390/ijerph19074044 (PMC8997762; doi:10.3390/ijerph19074044)
Supplement: Supplementary file 1 [file ijerph-19-04044-s001.zip › ijerph-1581724-supplementary.pdf]

Supplementary: Questionnaires and guidance

Form 1: Case and control initial reporting form (Day 1)

Automatically generated by Go.Data or entered by interviewer

**1. Administration**	
Unique health worker ID	
Case/control status[1]	☐ Case ☐ Control
Name of health care facility	
Form completion date (dd/mm/yyyy)	___/___/___
Was the interviewer blind to the case/control status of the interviewee?	☐ Yes<br>☐ No<br>☐ Unknown

***Instruction 1**: personal information will be kept confidential by the local investigators according to their procedure. No personal information will be sent or shared in any way with WHO. No personal information will be published. When we publish the results of this study, your confidential personal information will not be shown.*

**2. Identifier and basic information**	
First name[2]	
Surname[2]	
Sex	☐ Male ☐ Female ☐ Not known
Date of birth (dd/mm/yyyy)[2]	___/___/___
Telephone (mobile) number[2]	
Age (years, months)	
Email address[2]	
National social number/identifier (if applicable)[2]	
Country of residence	
Nationality	
Occupation in health care facility	☐ Medical doctor<br>☐ Registered nurse (or equivalent)<br>☐ Assistant nurse, nurse technician (or equivalent)<br>☐ Radiology/x-ray technician<br>☐ Phlebotomist<br>☐ Physical therapist<br>☐ Nutritionist/dietician<br>***Other health provider***:<br>☐ Laboratory personnel<br>☐ Admission/reception clerk<br>☐ Patient transporter<br>☐ Catering staff<br>☐ Cleaner

[1] To be filled in by the lead investigator after the interview to ensure that the interviewer is blind to the case/control status of the interviewee.
[2] All of these variables will be anonymized.

	☐ Administration/clerk<br>☐ Other [***specify***]:
Educational level	☐ None<br>☐ Primary<br>☐ Secondary<br>☐ Tertiary/University<br>☐ Prefer not to answer

***Instruction 2**: The information collected below will not be linked to your confidential personal information. Please answer the questions below honestly. Your data will help us to understand the potential risk factors for SARS-CoV-2 infection among health workers and to prevent future infections of health workers.*

**3. Context**	
Are you a health worker specifically dedicated to caring for COVID-19 patients?	☐ Yes<br><br>If yes, please specify the number of days dedicated to COVID-19 patients only during previous 14 days: _________<br><br>☐ No<br>☐ There are no COVID-19 dedicated staff in my facility
Did you receive specific training in the care of COVID-19 patients?	☐ Yes<br>☐ No<br>☐ Not sure
In the past 14 days, outside of your occupational duties have you been in contact with a person or persons known to have been diagnosed with Covid-19?	☐ Yes<br><br>If yes, was this<br>☐ household<br>☐ professional colleagues<br>☐ Most days (≥ 8 days)<br>☐ Some days (4–7 days)<br>☐ Few days (≤ 3 days)<br><br>☐ everyday social interactions (e.g. public transport, market)<br>☐ No<br>☐ Not sure
In the past 14 days, how often have you used public transport?	☐ Most days (≥ 8 days)<br>☐ Some days (4–7 days)<br>☐ Few days (≤ 3 days)<br>☐ Not used public transport
In the past 14 days, how often have you had social interaction with individuals outside of work, home or transport (e.g. in markets, shops etc.)	☐ Most days (≥ 8 days)<br>☐ Some days (4–7 days)<br>☐ Few days (≤ 3 days)<br>☐ Not had any other social interaction

4. Adherence to infection prevention and control (IPC) measures	
What was the date of your most recent IPC training within the health care facility (dd/mm/yyyy)?	___/___/___<br>☐ Forgotten/not sure<br>☐ I don't know what IPC standard precautions are
How much cumulative IPC training (standard precautions, additional precautions) have you received at this health care facility?	☐ Less than 2 hours<br>☐ More than 2 hours<br>☐ I don't know what IPC standard precautions are
Was the IPC training on personal protective equipment (PPE) carried out remotely (e.g. presentations only, e-learning) or were practical sessions on standard precautions/additional precautions conducted?	☐ Only remotely/theoretical<br>☐ Only practical<br>☐ Both<br>☐ I don't know what IPC standard precautions are
Do you know the recommended moments for hand hygiene in health care?	☐ I don't know them<br>☐ Yes, all 3<br>☐ Yes, all 4<br>☐ Yes, all 5<br>☐ Yes, all 6
Do you follow recommended hand hygiene practices?	☐ Always, as recommended<br>☐ Most of the time<br>☐ Occasionally<br>☐ Rarely<br>☐ Never
Do you use alcohol-based hand rub or soap and water before touching a patient?	☐ Always, as recommended<br>☐ Most of the time<br>☐ Occasionally<br>☐ Rarely<br>☐ Never
Do you use alcohol-based hand rub or soap and water before cleaning/aseptic procedures?	☐ Always, as recommended<br>☐ Most of the time<br>☐ Occasionally<br>☐ Rarely<br>☐ Never
Do you use alcohol-based hand rub or soap and water after (risk of) body fluid exposure?	☐ Always, as recommended<br>☐ Most of the time<br>☐ Occasionally<br>☐ Rarely<br>☐ Never
Do you use alcohol-based hand rub or soap and water after touching a patient?	☐ Always, as recommended<br>☐ Most of the time<br>☐ Occasionally<br>☐ Rarely<br>☐ Never
Do you use alcohol-based hand rub or soap and water after touching a patient's surroundings?	☐ Always, as recommended<br>☐ Most of the time<br>☐ Occasionally<br>☐ Rarely
Is alcohol-based hand rub available at point of care	☐ Yes<br>☐ No<br>☐ Occasionally<br>☐ Not sure

Do you follow IPC standard precautions when in contact with any patient?	☐ Always, as recommended<br>☐ Most of the time<br>☐ Occasionally<br>☐ Rarely<br>☐ Never<br>☐ I don't know what IPC standard precautions are
Do you wear PPE when indicated?<br><br>(PPE includes: medical/surgical mask, face shield, gloves, goggles/glasses, gown, coverall, head cover, respirator (e.g. N95 or equivalent) and shoe covers)	☐ Always, according to the risk assessment<br>☐ Most of the time, according to the risk assessment<br>☐ Occasionally<br>☐ Rarely<br>☐ Never
Is PPE available in sufficient quantity in the health care facility?	☐ Yes ☐ No ☐ Unknown
If no, which PPE is missing?	☐ Medical/surgical masks<br>☐ Face shield or goggles/glasses<br>☐ Gloves<br>☐ Gown and coverall<br>☐ Head cover<br>☐ Respirator (e.g. N95, FFP2 or equivalent) used when exposed to aerosol-generating procedures<br>☐ Shoe covers

**5. Exposures to COVID-19 infected patient(s)**	
Date of admission of COVID-19 confirmed patient (dd/mm/yyyy)<br><br>(If you were exposed to more than one COVID-19 patient, please provide the earliest admission date among them)	___/___/___<br><br>☐ Unknown
How many COVID-19 patients have you been exposed to during your occupational duties?	__________ (a range is also permissible)
Have you had close contact (within 1 metre) with the patient(s) since their admission?	☐ Yes ☐ No ☐ Unknown
• If yes, how many times (total)?	☐ < 10 times<br>☐ 10–50 times<br>☐ > 50 times<br><br>Please specify exactly how many times (if you can recall, optional): __________
• If yes, what was the maximum amount of time you spent with a COVID-19 patient?	☐ < 5 minutes<br>☐ 5–15 minutes<br>☐ > 15 minutes
• If yes, did you have prolonged face-to-face exposure (> 15 minutes)?	☐ Yes ☐ No ☐ Unknown<br><br>If yes, did you wear PPE?<br>☐ Yes ☐ No ☐ Unknown

	If yes, what type?<br>Tick all that apply:<br>☐ Medical/surgical mask<br>☐ Face shield<br>☐ Gloves<br>☐ Goggles/glasses<br>☐ Gown<br>☐ Coverall<br>☐ Head cover<br>☐ Respirator (e.g. N95, FFP2 or equivalent)<br>☐ Shoe covers
- If you were wearing a medical/surgical mask, what type?	
- If you were wearing a respirator, was it test fitted?	☐ Yes ☐ No ☐ Unknown
- If you were wearing gloves, did you remove them after contact with the patient?	☐ Yes ☐ No ☐ Unknown
• If yes, did you perform hand hygiene before contact with the patient?	☐ Always, as recommended<br>☐ Most of the time<br>☐ Occasionally<br>☐ Rarely<br>☐ Never<br><br>If yes:<br>☐ Alcohol-based hand rub<br>☐ Soap and water<br>☐ Water
• If yes, did you perform hand hygiene after contact with the patient?	☐ Always, as recommended<br>☐ Most of the time<br>☐ Occasionally<br>☐ Rarely<br>☐ Never<br><br>If yes:<br>☐ Alcohol-based hand rub<br>☐ Soap and water<br>☐ Water
• If yes, were you present for any aerosolizing procedures performed on the patient?	☐ Yes ☐ No ☐ Unknown<br><br>If yes, describe the procedure:<br><br>If yes, did you wear PPE?<br>☐ Yes ☐ No ☐ Unknown<br><br>If yes, what type?<br>Tick all that apply:<br>☐ Medical/surgical mask<br>☐ Face shield<br>☐ Gloves<br>☐ Goggles/glasses

	☐ Gown<br>☐ Coverall<br>☐ Head cover<br>☐ Respirator (e.g. N95, FFP2 or equivalent)<br>☐ Shoe covers
• If yes, did you come into contact with the patient's body fluids?	☐ Yes ☐ No ☐ Unknown<br><br>If yes, which body fluids:__________<br><br>If yes, were you wearing PPE?<br>☐ Yes ☐ No ☐ Unknown<br><br>If yes, what type?<br>Tick all that apply:<br>☐ Medical/surgical mask<br>☐ Face shield<br>☐ Gloves<br>☐ Goggles/glasses<br>☐ Gown<br>☐ Coverall<br>☐ Head cover<br>☐ Respirator (e.g. N95, FFP2 or equivalent)<br>☐ Shoe covers
Have you had direct contact with the patient's materials since their admission?<br><br>(Patient's materials include personal belongings, linen and medical equipment that the patient may have come into contact with)	☐ Yes ☐ No ☐ Unknown
• If yes, which materials?	Tick all that apply:<br>☐ Clothes<br>☐ Personal items<br>☐ Linen<br>☐ Medical devices used on the patient<br>☐ Medical equipment connected to the patient (e.g. ventilator, infusion pump etc.)<br>☐ Other: __________
• If yes, how many times since their admission (total)?	☐ < 10 times<br>☐ 10–50 times<br>☐ > 50 times<br><br>Please specify exactly how many times (if you can recall, optional): __________
• If yes, did you come into contact with the patient's body fluids via the patient's materials?	☐ Yes ☐ No ☐ Unknown<br><br>If yes, which body fluids: __________<br>If yes, were you wearing PPE?

	☐ Yes ☐ No ☐ Unknown<br><br>If yes, what type?<br>Tick all that apply:<br>☐ Medical/surgical mask<br>☐ Face shield<br>☐ Gloves<br>☐ Goggles/glasses<br>☐ Gown<br>☐ Coverall<br>☐ Head cover<br>☐ Respirator (e.g. N95, FFP2 or equivalent)<br>☐ Shoe covers
• If yes, did you perform hand hygiene before coming into contact with the patient's materials?	☐ Always, as recommended<br>☐ Most of the time<br>☐ Occasionally<br>☐ Rarely<br>☐ Never<br><br>If yes:<br>☐ Alcohol-based hand rub<br>☐ Soap and water<br>☐ Water
• If you were wearing gloves, did you remove them after contact with the patient?	☐ Yes ☐ No ☐ Unknown
• If yes, did you perform hand hygiene after contact with the patient's materials?	☐ Always, as recommended<br>☐ Most of the time<br>☐ Occasionally<br>☐ Rarely<br>☐ Never<br><br>If yes:<br>☐ Alcohol-based hand rub<br>☐ Soap and water<br>☐ Water
Have you had direct contact with the surfaces around the patient?	☐ Yes ☐ No ☐ Unknown
• If yes, which surfaces?	Tick all that apply:<br>☐ Bed<br>☐ Bathroom<br>☐ Ward corridor<br>☐ Patient table<br>☐ Bedside table<br>☐ Dining table<br>☐ Medical gas panel<br>☐ Other: __________
• How many times since their admission (total)?	☐ < 10 times<br>☐ 10–50 times<br>☐ > 50 times<br>Please specify exactly how many times (if

	you can recall, optional): __________
• If yes, did you come into contact with the patient's body fluids via the surfaces around the patient?	☐ Yes ☐ No ☐ Unknown<br><br>If yes, which body fluids: __________<br><br>If yes, were you wearing PPE?<br>☐ Yes ☐ No ☐ Unknown<br><br>If yes, what type?<br>Tick all that apply:<br>☐ Medical/surgical mask<br>☐ Face shield<br>☐ Gloves<br>☐ Goggles/glasses<br>☐ Gown<br>☐ Coverall<br>☐ Head cover<br>☐ Respirator (e.g. N95, FFP2 or equivalent)<br>☐ Shoe covers
• If yes, did you perform hand hygiene after contact with these surfaces?	☐ Always, as recommended<br>☐ Most of the time<br>☐ Occasionally<br>☐ Rarely<br>☐ Never<br><br>If yes:<br>☐ Alcohol-based hand rub<br>☐ Soap and water<br>☐ Water

**6a. Health worker symptoms**	
Date of first symptom onset (dd/mm/yyyy)	___/___/___<br>☐ Forgotten/not sure
Fever (≥ 38 °C) or history of fever	☐ Yes ☐ No ☐ Unknown<br>If yes, specify maximum temperature:_______
**Respiratory symptoms:**	
Sore throat	☐ Yes ☐ No ☐ Unknown
Cough	☐ Yes ☐ No ☐ Unknown
Runny nose	☐ Yes ☐ No ☐ Unknown
Shortness of breath	☐ Yes ☐ No ☐ Unknown
**Other symptoms:**	
Chills	☐ Yes ☐ No ☐ Unknown
Vomiting	☐ Yes ☐ No ☐ Unknown
Nausea	☐ Yes ☐ No ☐ Unknown
Diarrhoea	☐ Yes ☐ No ☐ Unknown
Headache	☐ Yes ☐ No ☐ Unknown
Rash	☐ Yes ☐ No ☐ Unknown
Conjunctivitis	☐ Yes ☐ No ☐ Unknown
Muscle ache	☐ Yes ☐ No ☐ Unknown

Joint ache	☐ Yes ☐ No ☐ Unknown
Loss of appetite	☐ Yes ☐ No ☐ Unknown
Loss of smell (anosmia) or taste	☐ Yes ☐ No ☐ Unknown
Nosebleed	☐ Yes ☐ No ☐ Unknown
Fatigue	☐ Yes ☐ No ☐ Unknown
Seizures	☐ Yes ☐ No ☐ Unknown
Altered consciousness	☐ Yes ☐ No ☐ Unknown
Other neurological signs	☐ Yes ☐ No ☐ Unknown<br>If yes, specify: __________
Other symptoms	☐ Yes ☐ No ☐ Unknown<br>If yes, specify: __________

**6.b Radiology report**	
Have you had radiological evidence of pneumonia (e.g. by chest X-ray or computed tomography scan) since the patient was admitted?	☐ Yes ☐ No ☐ Unknown

**7. Health worker pre-existing condition(s)**	
Do you have any underlying disease or pre-existing condition(s)?	☐ Pregnancy<br>If yes, specify trimester:<br>☐ First ☐ Second ☐ Third ☐ Unknown<br>☐ Obesity<br>If yes, BMI: _______<br>☐ Cancer<br>☐ Diabetes<br>☐ HIV/other immune deficiency<br>☐ Heart disease<br>☐ Asthma (requiring medication)<br>☐ Chronic lung disease (non-asthma)<br>☐ Chronic liver disease<br>☐ Chronic haematological disorder<br>☐ Chronic kidney disease<br>☐ Chronic neurological impairment/disease<br>☐ Organ/bone marrow recipient<br>☐ No<br>☐ Unknown<br>☐ Others, please specify: _________________

**8. Treatment/medications(s)**	
Are you taking any medication(s) regularly (apart from those for COVID-19)?	☐ Statin medication<br>☐ Steroid medication<br>☐ Antidiabetic medication<br>☐ Immunosuppressive medication<br>☐ No<br>☐ Unknown<br>☐ Other(s), please specify: _________________

Did you receive a prophylactic treatment for COVID-19 in the last 14 days?	☐ Yes ☐ No ☐ Unknown<br><br>If yes, which drug was received: _________________<br><br>Date started (dd/mm/yyyy) ___/___/___<br><br>Date stopped (dd/mm/yyyy) ___/___/___<br><br>Dosage: _________
